# Effects of dietary chromium picolinate supplementation on broiler growth performance: A meta-analysis

**DOI:** 10.1371/journal.pone.0249527

**Published:** 2021-04-06

**Authors:** Chao Feng, Qiqige Wuren, Xinyu Zhang, Xiaoying Sun, Qin Na

**Affiliations:** 1 Department of Life Science, Hulunbuir University, Hulunbuir, Inner Mongolia Autonomous Region, China; 2 Intelligent Agriculture and Animal Husbandry Academician Workstation, Hulunbuir University, Hulunbuir, Inner Mongolia Autonomous Region, China; 3 Department of Agriculture and Forestry, Hulunbuir University, Hulunbuir, Inner Mongolia Autonomous Region, China; Tokat Gaziosmanpasa Universitesi, TURKEY

## Abstract

**Objective:**

A meta-analysis was conducted to assess the effects of dietary chromium picolinate (CrPic) supplementation on broiler growth performance and to determine whether such effects are regulated by broiler strains, sex, environmental stress, or contextual factors including study area and years.

**Methods:**

Eligible studies were identified by searching the Web of Science, Springer, Elsevier, ScienceDirect, Taylor & Francis Online databases. Weighted average differences with corresponding 95% confidence intervals were computed with a random-effects model. We performed subgroup analysis stratified by study area, published years, broiler strains and sex, and environmental stress. Publication bias was assessed with Egger’s test method. A total of 15 studies eligible for inclusion.

**Results:**

The results indicated that CrPic supplementation significantly improved broiler growth performance and subgroup analysis confirmed this conclusion. We also found that Ross 308 or male broilers might be more sensitive to CrPic supplementation and showed better growth performance. A model was used to obtain the amount of chromium addition under the optimal growth performance, which suggested that the maximum value of average daily gain (ADG) was reached when chromium addition was 1810 μg/kg. The results of the sensitivity analysis showed low sensitivity and high stability of the meta-analysis.

**Conclusions:**

CrPic supplementation had a positive effect on the growth performance of broilers, and this meta-analysis provides a more accurate value of chromium addition, which may be beneficial for the practice of the broiler industry.

## Introduction

Glucose metabolism in birds differs considerably from mammals as birds maintain higher blood glucose concentrations and lower insulin levels than other vertebrates of similar body mass [1]. Birds are considered to be less sensitive to insulin compared with mammals, and the effect of chromium on enhancing insulin action and promoting efficiency of glucose, protein and fat metabolism are well documented [2, 3]. Besides, chromium has been shown to be active in rats, mice and domestic livestock, which seem to be related either to an apparent shifting of energy partitioning or an improved immunological competence and stress resistance [4, 5].

Chromium usually exists in the form of inorganic compounds or organic complexes [4]. The most common oxidation states of chromium are the metallic form (Cr^0^), trivalent form (Cr^3+^), and hexavalent form (Cr^6+^), in which Cr^3+^ is the most stable state and highly safe form [6]. Organic chromium, usually found as Cr-picolinate, Cr-methionine, Cr-nicotinic acid complex, and Cr-yeast is considered to have higher bioavailability and absorption rates than inorganic chromium [7, 8]. In general, inorganic chromium’s bioavailability is between 1% and 3% while organic chromium can be up to 15% to 30% [9]. This can be explained by the chelation of the mineral with organic acids, amino acids, peptides or other compounds [4]. The legality of adding supplemental chromium to animal diets varies among countries and animal species [10]. Chromium is not authorized as a feed additive in the European Union to avoid additional exposure of consumers resulting from the use of supplemental chromium in animal nutrition [11]. In the United States, Food and Drug Administration (FDA) permission or approval is required for any chromium source to be supplemented to animal diets [10]. In 2016, the FDA approved the use of chromium (200 ppb) in complete feed for broiler chickens; however, there are still no National Research Council (NRC) recommendations for chromium in broiler diets [12]. The Standardization Administration of China also has no regulations on chromium supplementation in the latest national standards of formula feeds for layers and broilers [13].

Organic chromium in the form of chromium picolinate (CrPic) was often used as a feed additive in previous studies involving the effect of chromium on the broiler growth performance [2, 14]. Some researchers found broilers fed supplemental CrPic showed a positive effect on growth performance [15, 16], and the beneficial effects of chromium can be observed more efficiently under environmental, dietary and hormonal stress [17, 18]. Sahin et al. revealed that increased supplemental chromium (200 to 1200 μg/kg CrPic) increased body weight and feed intake [19]. Huang et al. suggested that birds fed 400 or 2000 μg/kg CrPic could promote the average daily gain of broilers and improve the carcass traits and meat quality of broilers under heat stress conditions [17]. However, some studies showed the opposite, suggesting that CrPic supplementation did not affect broiler growth performance. Kim et al. indicated that dietary addition of chromium (100 to 800 μg/kg CrPic) did not affect body weight, feed intake or feed conversion (1). Indeed, studies showed that chromium supplementation could improve broiler growth performance under heat stress conditions, but no such effect existed under normal conditions [14, 17, 20, 21]. Zheng et al. believed that heat stress conditions were the critical factor [14].

However, such effects on broiler growth performance might be inconsistent. Akbari and Torki found that broiler growth performance was not influenced by dietary chromium supplementation (1000 μg/kg CrPic) [22]. According to Zha et al., CrPic significantly increased broiler body weight and feed efficiency, but CrCl_3_ did not under heat stress conditions [20]. Thus, the effect of chromium supplementation on broiler growth performance has not been determined, especially for chromium picolinate, which has been widely used in the poultry industry.

Meta-analysis is a kind of statistical method that integrates research results of all types in the same field. The differences between studies were removed by meta-analysis, making the corrected data comparable, creating more objective and convincing conclusions [23]. The main goal of this study is to undertake a meta-analysis of trials assessing the effect of CrPic supplementation on broiler growth performance, obtain a reasonable quantitative model to explain the observed value and provide a theoretical reference for the practical process. Specifically, we hypothesized that chromium picolinate supplementation could significantly improve the broiler growth performance; however, the addition might be higher than FDA recommendations for chromium, and environmental stress conditions might affect the growth performance of broiler to chromium supplementation.

## Materials and methods

### Study selection

All kinds of studies published from January 1990 to November 2020 were retrieved from the Web of Science, Springer, Elsevier, ScienceDirect, and Taylor & Francis Online databases. The following search terms were used (broiler OR chick*) AND (performance OR growth) AND (chromium picolinate or CrPic), and 1330 records were identified through database searching. Titles and abstracts of all potentially relevant publications were rigorously reviewed to assess their relevance to the study, and the full text was further scrutinized if any potentially relevant information was identified during the process.

The selected studies met the following criteria: published in English, used a corn and soybean meal-based diet, had a 42-day experimental period, provided the specific CrPic addition values, and pertained to nonindigenous strains. Newborn broilers were divided into groups based on the CrPic supply they received. The addition of Cr in the control group was 0 mg/kg, while that in the experimental group ranged from 0.1 to 3.2 mg/kg. During the 42-day experimental period, the selected studies in our study recorded the weight changes and food intake of broilers daily and finally calculated the average daily gain (ADG), average daily feed intake (ADFI) and feed conversion ratio (FCR). The data in these studies included means and variances and encompassed at least three independent replicates of each treatment.

### Data extraction

Under the standardized data-collection protocol, the following data were extracted from each study: author’s name, country, publication year, broiler strains, broiler sex, feed ingredients, CrPic addition, environmental stress, sample size, and experimental periods. We extracted the means of ADG, ADFI and FCR in both the control and experimental treatments, as well as the standard deviations (SD) and sample size (n). When the standard error (SE) was reported, we transformed it to SD by using the formula SD = SE * sqrt (n). If the data were presented graphically, we extracted data points through GetData software (http://www.getdata-graph-digitizer.com/). Data abstraction was performed independently by two reviewers, and any discrepancies were decided by the third reviewer or discussed to reach a consensus.

### Statistical analysis

The weighted mean difference (WMD) is used to estimate the effect size of the combined study. This method used the pooled effect estimate to represent a weighted average of all included study group comparisons, where the weighting assigned to each study group comparison result was inversely proportional to the variance. This method assigns more weight in the meta-analysis to larger trials and less weight to smaller trials [24]. A random-effects model taking into account both within- and between-study variation was used to compute the summary risk estimates [25]. We combined the data from included studies, and WMDs with the 95% confidence interval (CI) of a total change in ADG, ADFI and FCR were reported, which was considered as the measurements of the effect in this meta-analysis. Stratified analysis by geographic area, publication year, broiler strains and sex was also carried out. To assess the effects of each study and to verify the stability of the results of the meta-analysis, a sensitivity analysis was also conducted by omitting each study in turn and estimating the overall effects of the remaining studies sequentially. Statistical heterogeneity was assessed with Q and I^2^ statistics [26]. Potential publication bias was evaluated by using Egger’s test [27].

The PROC MIXED model (SAS Version 9.4; SAS Institute, Cary, NC) was used to analyze the relationship between organic chromium addition and broiler growth performance. The model is as follows:

$$Y_{ij}=B_{0}+B_{1}X_{ij}+B_{2}X_{ij}^{2}+S_{i}+b_{1i}X_{ij}+b_{2i}X_{ij}^{2}+e_{ij}$$

The same mixed linear model was used in a previously published meta-analysis and was explained in details by Feng et al (2020) [28]. The differences among the studies are assumed to be random effects. The intercept and slope of the variables (fixed effects) represent the average intercept and average slope of the ADG, ADFI, and FCR as they vary with dietary chromium additions in the mixed-effects model. The intercept and slope of the variables (random effects) represent essential factors not included in the different studies’ regression analyses [29]. The random effects of the y value are adjusted to remove differences between the studies, and then a regression analysis is performed to calculate the correlation coefficient (i.e., r^2^) [30]. The mixed-effects model code is shown below.

PROC MIXED data = data;

CLASS Group;

MODEL Y = X X*X/Solution OUTP = Predictionset OUTPM = PredY;

RANDOM intercept X X*X/TYPE = VC SUBJECT = Group SOLUTION;

RUN;

A regression analysis and curve fitting was performed for the relationship between the variables (y-axis) and chromium addition (x-axis). The calculation steps are as follows: (i) calculation of the residual, (ii) calculation of PredY, and (iii) determination of the adjusted y value for each independent variable (AdjuestedY = PredY + Residual) to remove the differences between the studies. All statistical analyses were performed using STATA (version 11.0; STATA Corp, College Station, TX) and SAS (Version 9.4; SAS Institute, Cary, NC) software.

## Results

### Study characteristics

A total of 15 studies that met our eligibility criteria were included in the meta-analyses [2, 14–22, 31–35] (Table 1). All eligible studies were published between 1995 and 2018, of which 26.7% were published in the 1990s, 33.3% in the 2000s and 40.0% in the 2010s. The study area is mainly distributed in Asia, including China, Korea, Iran, Turkey, Egypt (the only African country) and India, accounting for 40.0%, 20.0%, 13.3%, 13.3% 6.7% and 6.7%, respectively. In addition, we collected relevant information on broiler strains, basal diet, Cr addition, environmental stress, etc., and the details are listed in Table 1.

**Table 1:** Characteristics of the meta-analysis database.

Author	Country	Published year	Broiler strains	Sex	Basal diet	Cr source	Cr addition (μg/kg)	Sample size	Experimental period (d)	Heat stress
SW Kim	Korea	1995	Arbor Acres	Both	Corn + soybean	CrPic	0, 200	288	42	No
YH Kim	Korea	1996	Arbor Acres	Both	Corn + soybean	CrPic	0, 100, 200, 400, 600, 800	288	42	No
YH Kim	Korea	1996	Arbor Acres	Female	Corn + soybean	CrPic	0, 800, 1600, 2400	144	42	No
M Anandhi	India	2006	No report	Both	Corn + soybean	CrPic	0, 250, 500, 750	128	42	No
M. Akbari	Iran	2013	Cobb 500	Female	Corn + soybean	CrPic	0, 1000	240	42	Yes
S Ghanbari	Iran	2012	Ross 308	Male	Corn + soybean	CrPic	0, 400, 800, 1200, 1600, 2000	360	42	No
YL Huang	China	2015	Cobb 500	Female	Corn + soybean	CrPic	0, 400, 2000	252	42	Yes
DN Lee	China	2002	Avian	Both	Corn + soybean	CrPic	0, 200, 400, 800	320	42	No
TF Lien	China	1999	No report	Both	Corn + soybean	CrPic	0, 800, 1600, 3200	120	42	No
L Lu	China	2018	Arbor Acres	Male	Corn + soybean	CrPic	0, 400, 800, 800, 1200	240	42	No
K Sahin	Turkey	2002	Ross 308	Male	Corn + soybean	CrPic	0, 200, 400, 800, 1200	150	42	Yes
K Sahin	Turkey	2003	Ross 308	Male	Corn + soybean	CrPic	0, 400	120	42	Yes
SS Tawfeek	Egypt	2014	Cobb 500	Both	Corn + soybean	CrPic	0, 500	60	42	Yes
LY Zha	China	2009	Arbor Acres	Male	Corn + soybean	CrPic	0, 500	120	42	Yes
CC Zheng	China	2015	Cobb 500	Female	Corn + soybean	CrPic	0, 400, 2000	108	42	No

### Risk of bias in the included studies

**Fig 1:**
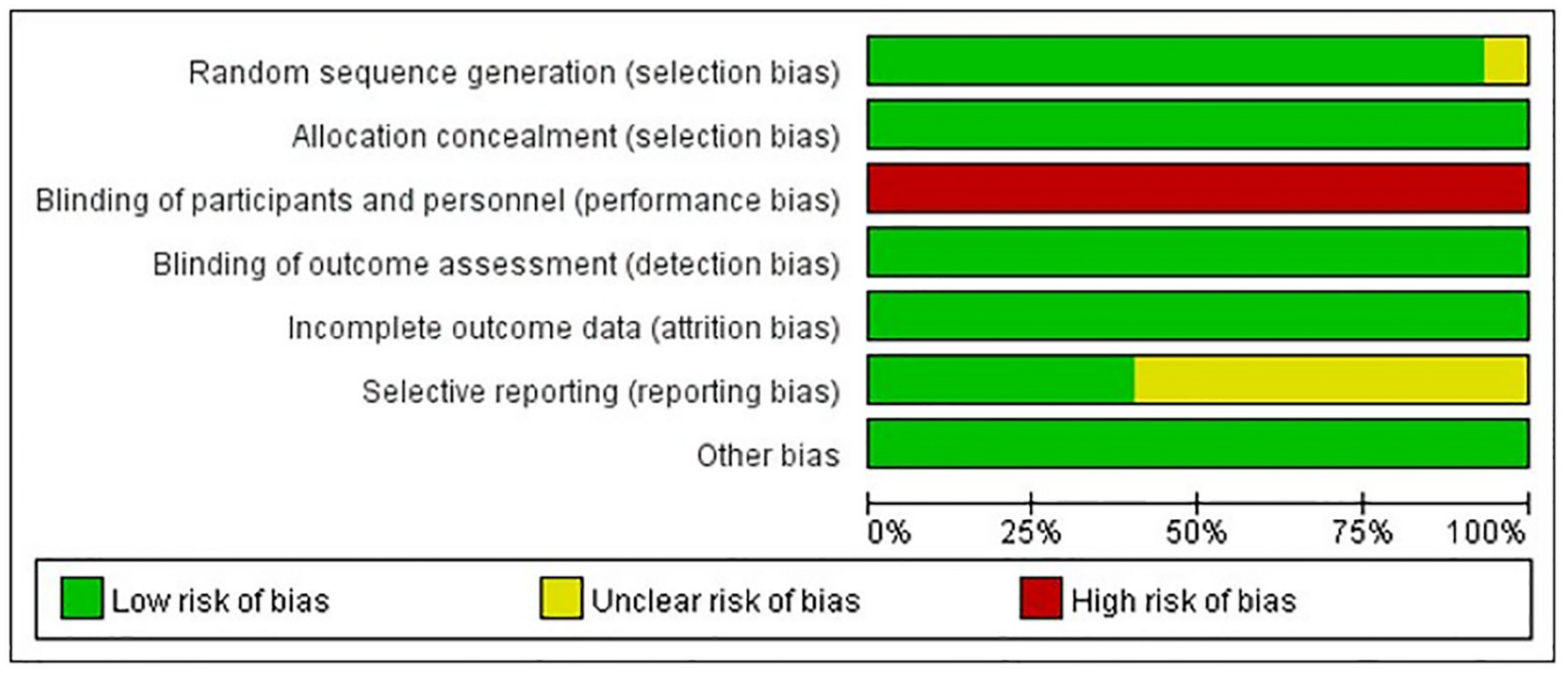
Risk of bias graph.

The summary and details of the risk of bias were summarized in Fig 1. All of the studies except one [15] described the randomization methods, and no studies were double-blinded; therefore, they remained at high risk of performance bias. Double blinding was avoided mostly because blinding was neither practical nor desirable in broiler growth experiment. No reporting bias was detected, while incomplete information was provided and many outcomes of potential importance were ignored for this domain in nine studies [2, 15, 16, 21, 22, 31–34]. There was no other apparent risk of bias in all included studies.

### Results of the meta-analysis and subgroup analysis

**Fig 2:**
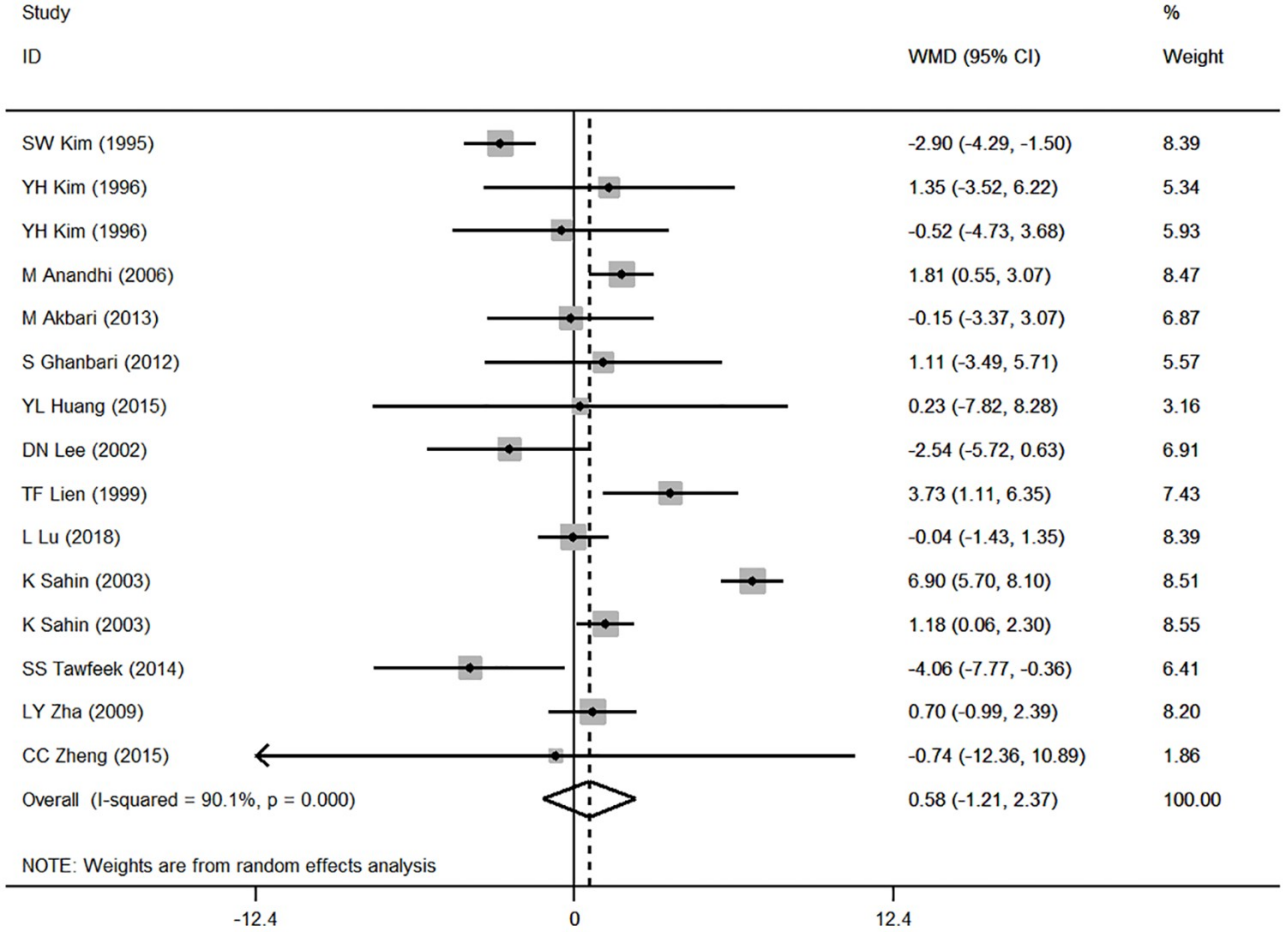
Forest plot of ADG.

**Fig 3:**
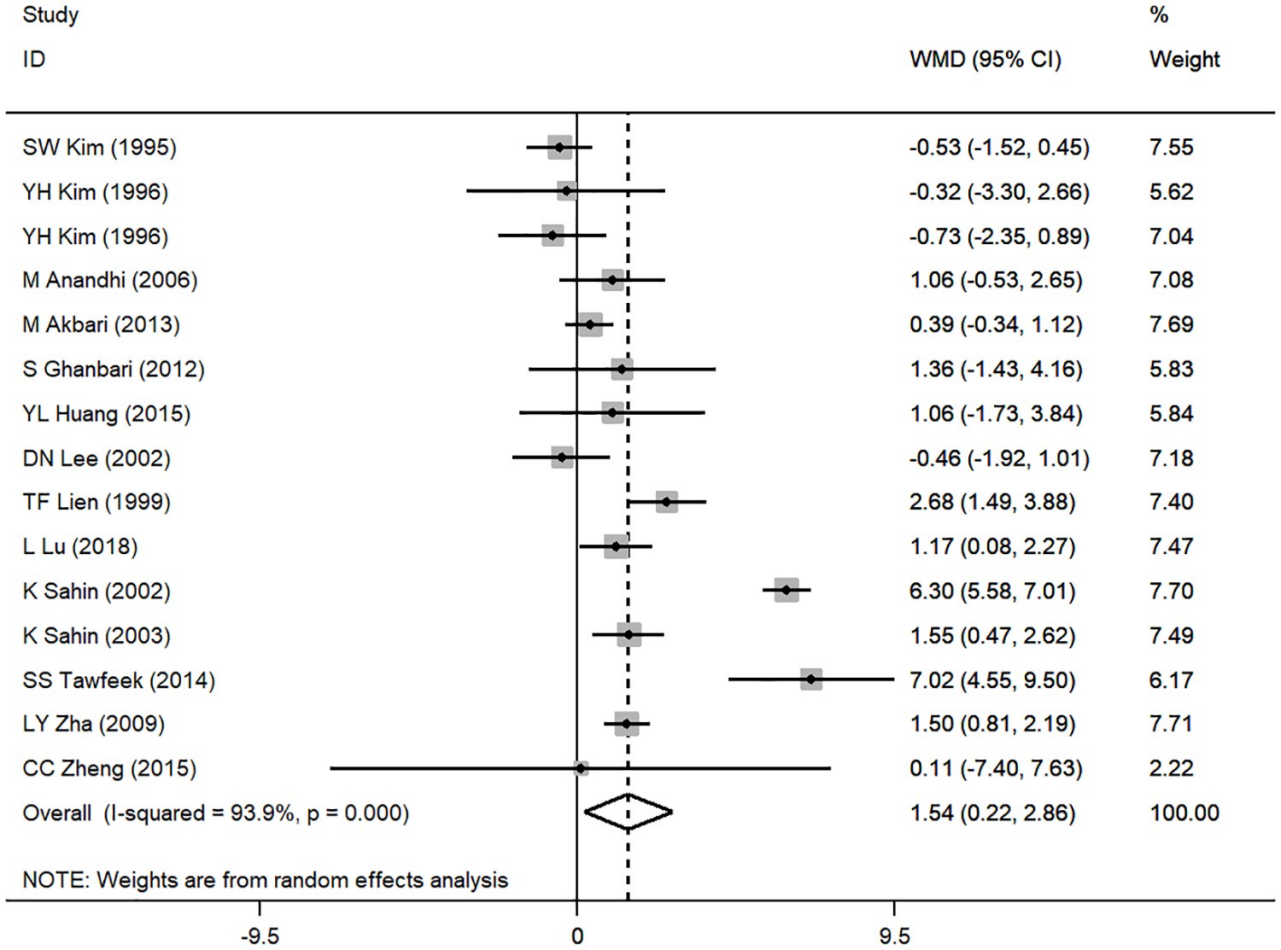
Forest plot of ADFI.

**Fig 4:**
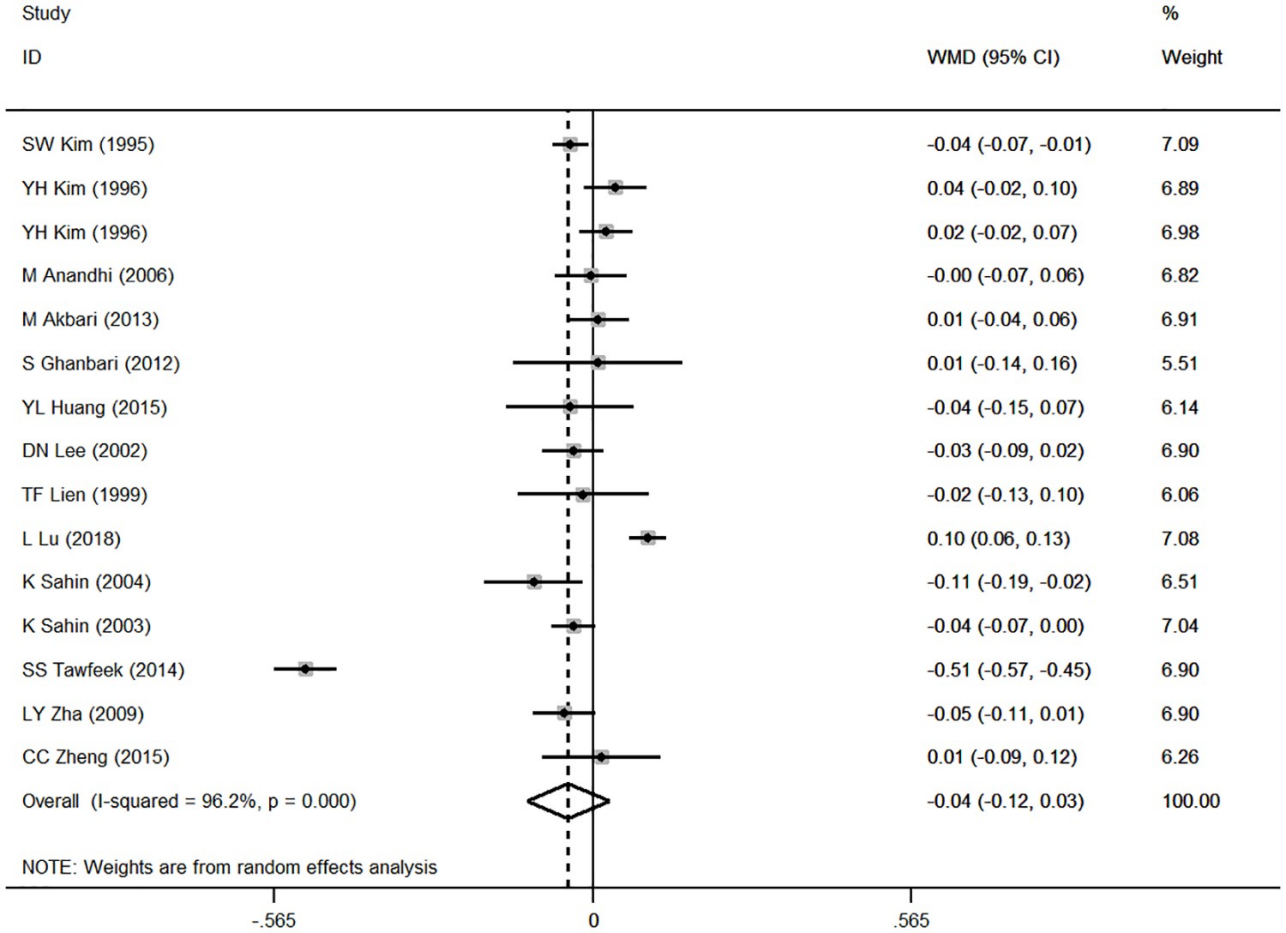
Forest plot of FCR.

Based on the results of the random effects method, the pooled WMDs of the ADG, ADFI and FCR were 1.543 (95% CI: 0.221 to 2.864), 0.580 (95% CI: -1.206 to 2.366), and -0.055 (95% CI: -0.126 to 0.017), respectively (Figs 2–4). The addition of CrPic to the diet had a significant effect on ADG (*P* = 0.022) but had no significant effect on ADFI or FCR (*P* = 0.524; *P* = 0.135) (Table 2). We detected significant heterogeneity in the studies (*P* < 0.001), with *I*^*2*^ values ranging from 83.9% to 96.2% (Table 2). In general, heterogeneity was still present but was attenuated in the ADG compared with the ADFI and FCR.

**Table 2:** Summary of weighted mean differences from meta-analyses and subgroup analysis by different factors.

	Stratification	No. of studies	Random effects model			Heterogeneity	
			WMD	95%CI	*P*	*I*^*2*^	*P*
**ADG**							
Main analysis		15	1.543	0.221–2.864	0.022	83.9%	<0.001
Country	Korea	3	-0.566	-1.376–0.245	0.171	0.0%	0.965
	India	1	–	–	–	–	–
	Iran	2	0.452	-0.254–1.158	0.209	0.0%	0.944
	China	6	1.280	0.414–2.146	0.004	54.4%	0.052
	Turkey	2	3.940	-0.715–8.595	0.097	98.1%	<0.001
	Egypt	1	–	–	–	–	–
Published year	1990s	4	0.352	-1.536–2.240	0.715	84.6%	<0.001
	2000s	5	2.027	-0.524–4.579	0.119	97.0%	<0.001
	2010s	6	1.929	0.206–3.653	0.045	80.5%	<0.001
Sex	Both	6	1.487	-0.395–3.368	0.121	88.6%	<0.001
	Female	4	0.247	-0.398–0.892	0.453	0.0%	<0.001
	Male	5	2.431	0.018–4.845	0.048	96.6%	<0.001
Broiler strains	Arbor Acres	5	0.384	-0.675–1.443	0.477	73.8%	0.004
	Cobb 500	4	2.384	-1.217–5.984	0.194	88.2%	<0.001
	Ross 308	3	3.170	-0.612–6.952	0.100	96.5%	<0.001
	Avian	1	–	–	–	–	–
	No report	2	1.958	0.376–3.540	0.015	61.0%	0.109
Heat stress	Yes	5	2.927	0.597–5.257	0.014	97.0%	<0.001
	No	10	0.556	-0.386–1.499	0.247	65.7%	<0.001
**ADFI**							
Main analysis		15	0.580	-1.206–2.366	0.524	90.1%	<0.001
Country	Korea	3	-1.531	-4.023–0.960	0.228	43.4%	0.171
	India	1	–	–	–	–	–
	Iran	2	0.264	-2.373–2.900	0.845	0.0%	0.660
	China	6	0.509	-1.118–2.136	0.540	50.2%	0.074
	Turkey	2	4.034	-1.571–9.640	0.158	97.9%	<0.001
	Egypt	1	–	–	–	–	–
Published year	1990s	4	0.307	-3.494–4.109	0.874	85.3%	<0.001
	2000s	5	1.782	-0.987–4.551	0.207	94.4%	<0.001
	2010s	6	-0.369	-1.519–0.781	0.529	0.0%	0.483
Sex	Both	6	-0.407	-3.040–2.227	0.762	87.3%	<0.001
	Female	4	-0.262	-2.645–2.122	0.830	0.0%	0.998
	Male	5	2.044	-0.933–5.020	0.178	94.6%	<0.001
Broiler strains	Arbor Acres	5	-0.555	-2.303–1.192	0.534	70.6%	0.009
	Cobb 500	4	-1.624	-3.904–0.657	0.163	0.0%	0.441
	Ross 308	3	3.240	-1.357–7.837	0.167	95.8%	<0.001
	Avian	1	–	–	–	–	–
	No report	2	2.415	0.669–4.162	0.007	40.3%	0.195
Heat stress	Yes	5	1.073	-2.113–4.258	0.509	93.4%	<0.001
	No	10	0.147	-1.617–1.911	0.870	77.4%	<0.001
**FCR**							
Main analysis		15	-0.055	-0.126–0.017	0.135	96.2%	<0.001
Country	Korea	3	0.004	-0.048–0.057	0.877	76.0%	0.016
	India	1	–	–	–	–	–
	Iran	2	-0.071	-0.124 –-0.019	0.007	0.0%	0.392
	China	6	-0.007	-0.076–0.061	0.838	87.0%	<0.001
	Turkey	2	-0.076	-0.112–0.040	<0.001	0.0%	0.475
	Egypt	1	–	–	–	–	–
Published year	1990s	4	0.001	-0.044–0.046	0.960	64.0%	0.040
	2000s	5	-0.051	-0.079 –-0.024	<0.001	17.9%	0.300
	2010s	6	-0.093	-0.295–0.110	0.370	98.5%	<0.001
Sex	Both	6	-0.095	-0.257–0.067	0.251	98.1%	<0.001
	Female	4	-0.029	-0.084–0.027	0.317	68.9%	0.022
	Male	5	-0.026	-0.117–0.065	0.576	92.6%	<0.001
Broiler strains	Arbor Acres	5	0.015	-0.046–0.076	0.637	90.3%	<0.001
	Cobb 500	4	-0.160	-0.406–0.085	0.201	98.3%	<0.001
	Ross 308	3	-0.072	-0.107 –-0.037	<0.001	0.0%	0.547
	Avian	1	–	–	–	–	–
	No report	2	-0.006	-0.062–0.049	0.823	0.0%	0.844
Heat stress	Yes	5	-0.146	-0.291 –-0.001	0.049	97.6%	<0.001
	No	10	0.011	-0.032–0.054	0.612	79.9%	<0.001

The subgroup analysis was stratified by country, published year, sex, broiler strains and heat stress. The pooled WMDs of ADG, ADFI and FCR in different subgroups are listed in Table 2. We found that the results of ADG in China or published in the 2010s and the results of FCR in Iran and Turkey or published in the 2000s had significant responses to chromium supplementation (Table 2). The male broilers and Ross 308 broilers showed obviously higher ADG and lower FCR, respectively (*P* = 0.048; *P* < 0.001). The ADG was higher in the male broiler or heat stress condition subgroup (*P* = 0.048; *P* = 0.014), and the FCR was lower in the Ross 308 or heat stress condition subgroup (*P* < 0.001; *P* = 0.049) (Table 2).

### Relationship between growth performance and CrPic

**Fig 5:**
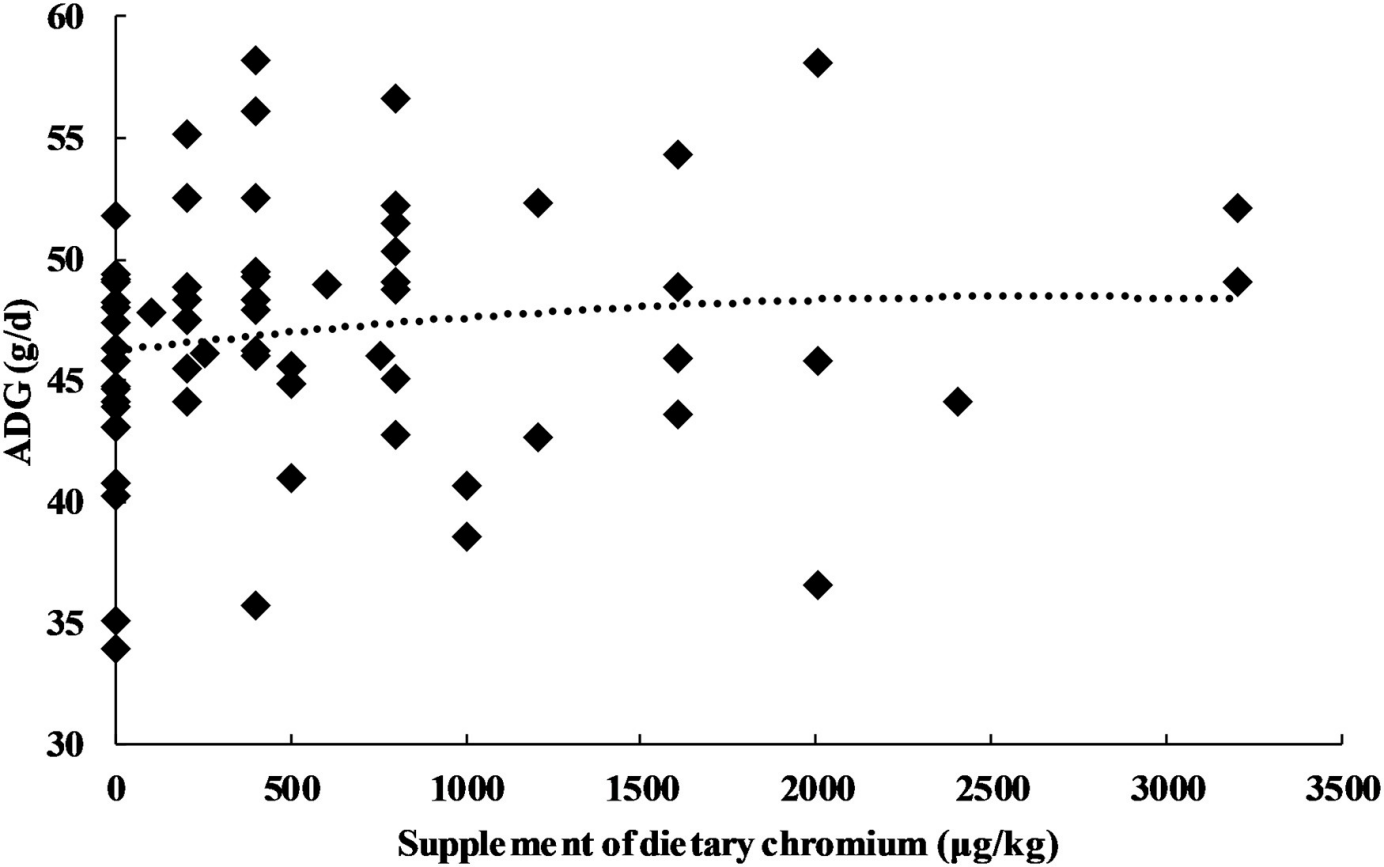
Relationship between chromium and adjusted ADFI.

Chromium addition was used as the independent variable, and the performance indices (ADG, ADFI and FCR) were used as the dependent variables. The results indicated a quadratic relationship between the adjusted ADG and chromium addition (Y_ADG_ = 44.816 + 3.512E-03X-9.721E-07X^2^, n = 66, *P* = 0.015). The maximum value of the ADG (48.010 g/d) was reached when chromium addition was 1.810 mg/kg (Fig 5). The test of fixed effects for the quadratic term was not significant at the 5% level in the MIXED procedure of ADG and FCR (*P* = 0.365 and 0.997, respectively).

### Sensitivity analysis and publication bias

**Fig 6:**
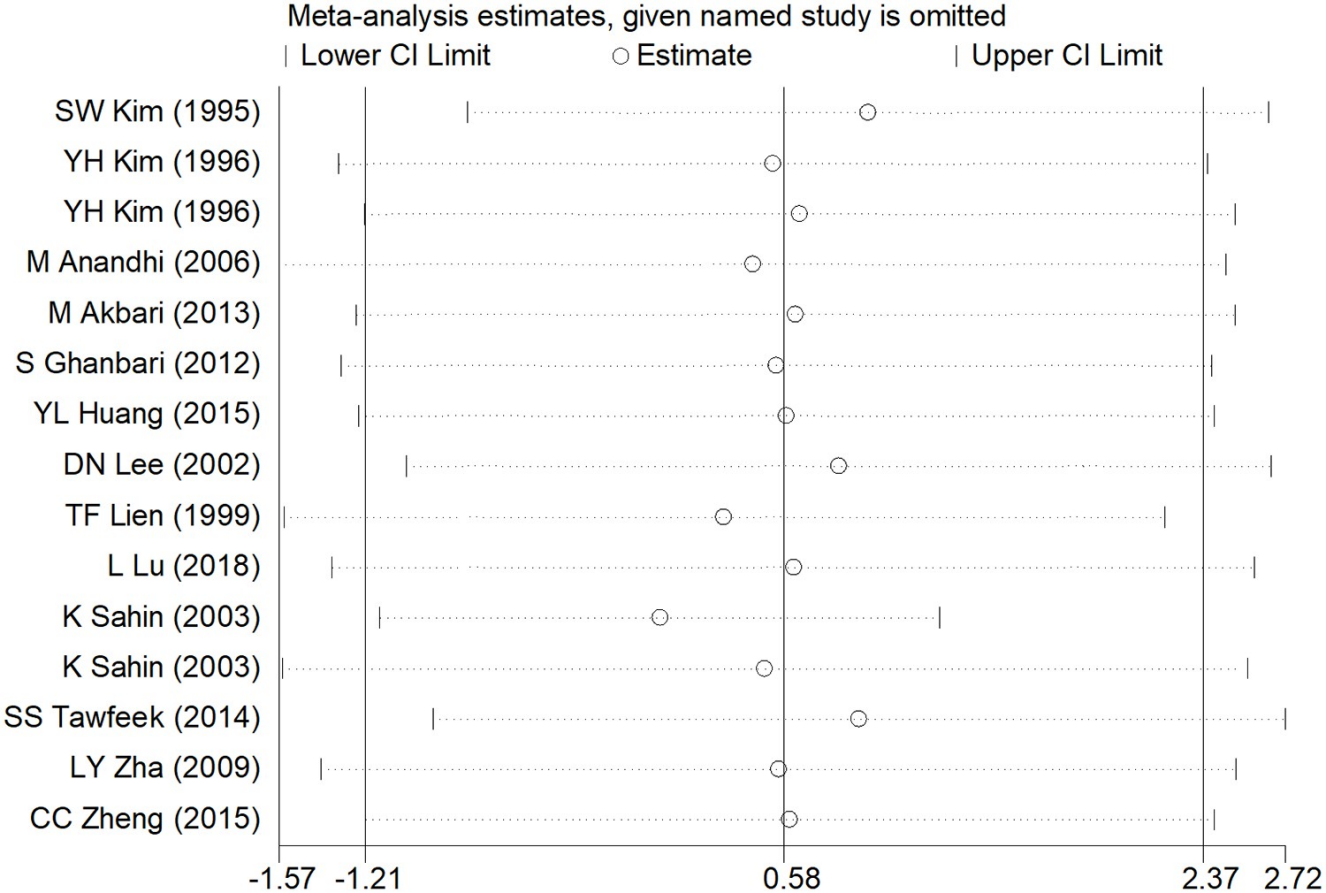
Sensitivity analysis of ADG.

**Fig 7:**
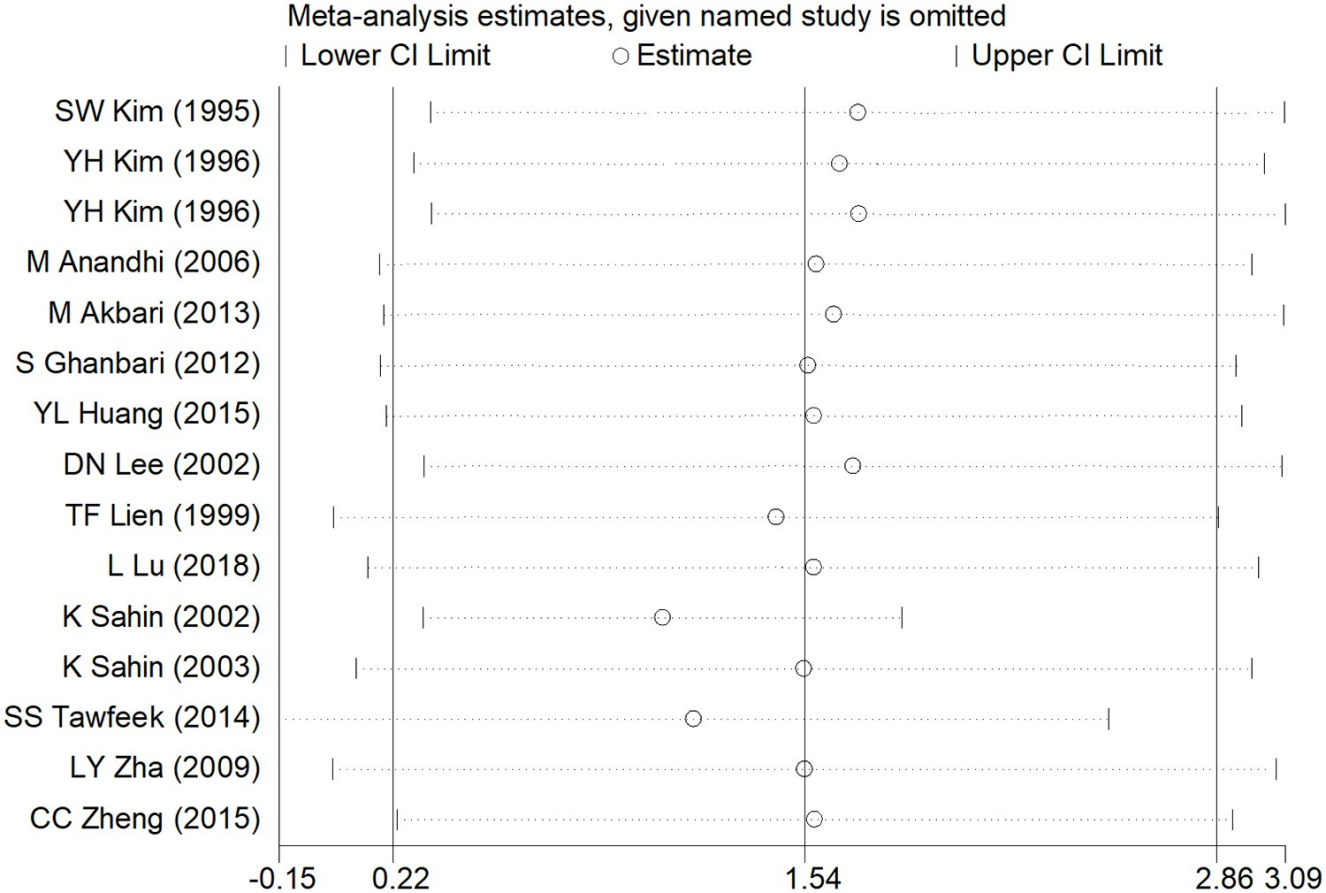
Sensitivity analysis of ADFI.

**Fig 8:**
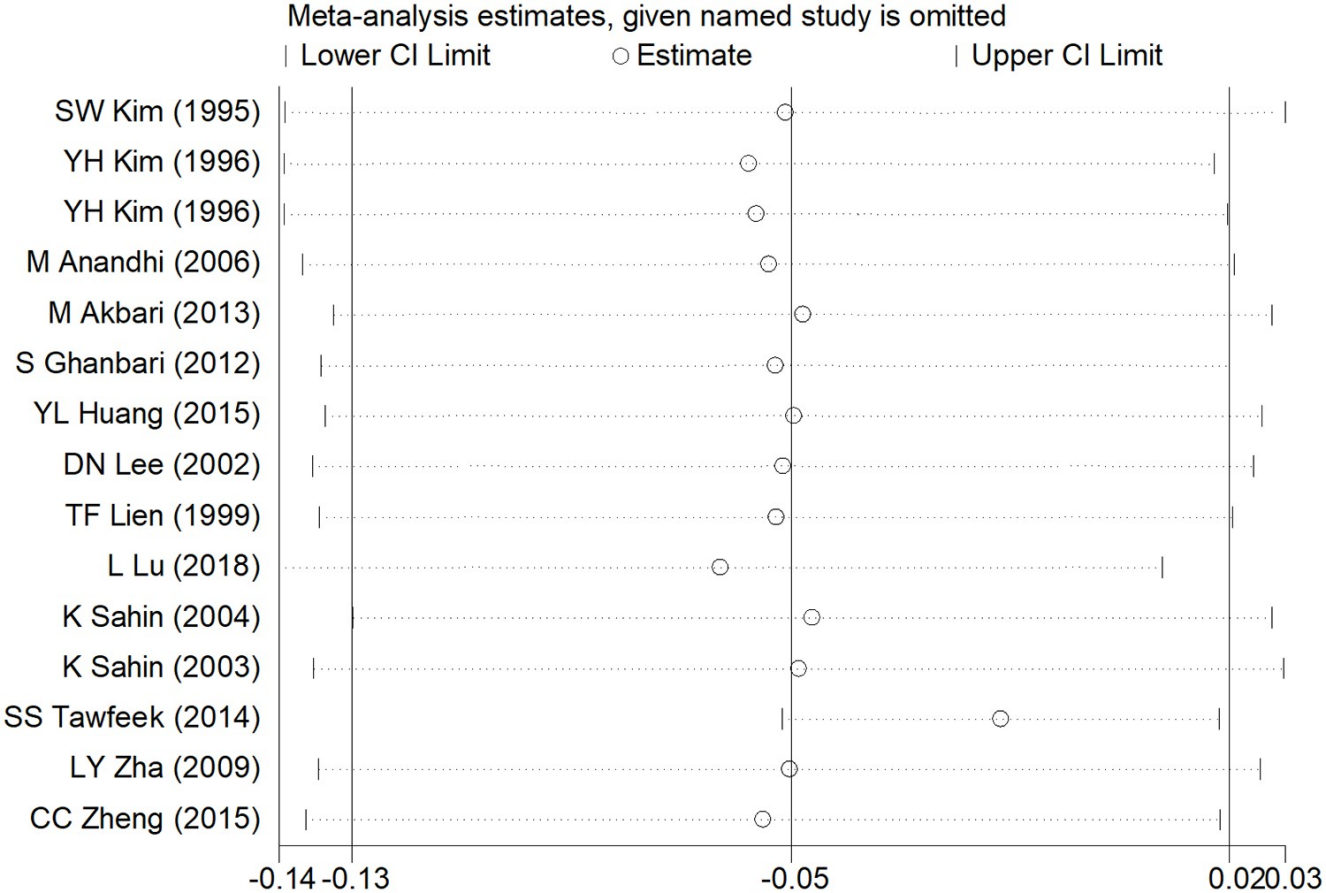
Sensitivity analysis of FCR.

After removing each study, the pooled estimates of the remaining studies were all located in the range of the overall effects, indicating that the meta-analysis results showed low sensitivity and high stability (Figs 6–8). Publication bias was assessed using Egger’s test. There was significant publication bias in ADG studies (t = 3.36, *P* = 0.005), while no significant publication bias existed in ADFI and FCR studies (t = 2.15, *P* = 0.052; t = -1.53, *P* = 0.150).

## Discussion

Chromium is considered an essential trace mineral for boilers in manipulating growth performance and improving carcass composition. However, there is still no recommended dosage of chromium supplementation in the broiler industry. In the United States, the Food and Drug Administration (FDA) approved the use of chromium propionate as the only organic source in broiler diets in 2016 and chromium is not authorized as a feed additive in the European Union [12]. NRC also did not specify the amount of chromium in poultry diets (NRC, 1997). Studies have reached different conclusions on the effect of CrPic addition on broiler growth performance. Some researchers suggested that CrPic could markedly increase body weight, feed intake and feed efficiency when supplementation was between 200 μg/kg and 1200 μg/kg [19, 20, 35]. However, studies also showed that dietary addition of CrPic might not affect growth performance and nutrient utilizability [2, 14, 31]. To our knowledge, the present study is the first meta-analysis of the association between organic chromium addition and broiler growth performance.

By analyzing the data from 15 studies, we found that adding CrPic significantly increased the ADG of broilers (Fig 2) but had no obvious effect on ADFI and FCR (Figs 3 and 4). The higher ADG in this study agreed with early reports [15–17], which may be attributed to the ability of chromium to regulate the level of glucose by activating insulin secretion and enabling proper metabolic transformations of carbohydrates, proteins and lipids. The ADFI and FCR results were in accord with studies that reported that CrPic supplementation had little effect on the growth performance of broilers[2, 14, 21, 33]. In other words, the effects of CrPic supplementation on broiler weight gain, food consumption, and feed conversion ratio are not consistent. The effects of Cr on the broilers’ growth performance are complex, and there is no clear conclusion [12]. We found that all studies showed significant improvement on ADG of broilers under heat stress conditions except for the study by Akbari and Torki. (1000 μg / kg Cr form CrPic) [22]. However, supplementation of CrPic under normal conditions showed inconsistent results [16, 21]. Therefore, we speculated that the reason might be related to environmental stress. Stressors such as high environmental temperature increase the production and release of corticosteroids, which affect glucose and mineral metabolism, hypercholesterolemia, gastrointestinal lesions, alterations in immune system functions, and decreased tissue vitamin and mineral concentrations in poultry as well as humans; however, chromium supplementation may decrease glucocorticoid release during stress [36]. In addition, other environmental stress such as cage area and unreported stress conditions might all greatly increase chromium mobilization from tissues and their excretion thus may exacerbate a deficiency or an increased requirement of chromium [3]. Compared with normal conditions, chromium supplementation under the environmental stress condition may significantly improve the growth performance, which resulted in inconsistent results for ADG, ADFI and FCR, as this meta-analysis included studies under both stress and normal conditions.

Compared with the overall analysis of ADG, our subgroup analysis produced more conservative effect estimates for the country and published year groups. CrPic addition was not associated with ADG in the subgroup of countries and published years except for China and the 2010s (Table 1). Although the overall analysis showed that CrPic addition had no pronounced effect on FCR, the subgroup analysis by country and publication year indicated an inverse association in Iran, Turkey and the 2000s (Table 1). We believe that the management mode and strategy of the broiler industry is developing and changing in different areas and periods, which may lead to the different use of chromium in the broiler industry and then affect the growth performance of broilers in different countries and published years. In addition, the subgroup analysis of ADG, ADFI and FCR stratified by published year was consistent with the overall analysis, which indicated the high stability of the meta-analysis results and was then confirmed by sensitivity analysis (Figs 6–8). We also found that male broilers had better growth performance than females (higher ADG), which suggested that males might be more sensitive to chromium addition. This result is consistent with our conclusion on the correlation between CrCl_3_ and broiler growth performance (unpublished data). Studies on growing-finishing pigs and sows showed that chromium increased lean body mass [37, 38]. The carcass lean rate of male broilers was always higher than that of females due to the different hormonal statuses, which may be the reason for the higher ADG of male broiler chickens. Besides, when both females and males were used in the studies, confounding effects of sex were likely to occur, leading to no significant response to chromium supplementation in “both” subgroups.

When stratified by broiler strains, Ross 308 showed better growth performance (lower FCR) than the other strains. The mechanism of improving the performance of Ross 308 by chromium addition may be related to its higher ability to acclimatize and adapt to new environmental conditions or dietary composition than the other strains [39]. In fact, these characteristics of broilers still cannot fully explain why broiler strains had different growth performances to CrPic. We believe that the different physiological responses of strains to chromium supply might be the fundamental reason, whereas the mechanism of these effects is still poorly understood. The feed cost constitutes 70% - 80% of the cost of raising broiler chickens; therefore, changes in FCR can have a major impact on the profitability of an operation [40]. Through subgroup analysis, we also found that chromium supplementation might promote growth performance under heat stress conditions (Table 1). Similar results have been demonstrated in previous studies on the influence of chromium under heat stress conditions [41, 42]. In contrast, some studies selected in our meta-analysis suggested no effect of dietary chromium supplementation on broiler growth performance under heat conditions [19, 22]. The lack of agreement among these studies may be partially explained by a combination of the following: (i) the concentration of supplemental chromium (ranging from 200 μg/kg to 2000 μg/kg) and (ii) the type of induced heat stress (constant temperature or cycling temperature ranging from 21.0°C to 38.3°C).

With the increase in chromium addition, the ADG of broilers showed a significant negative quadratic relationship, reaching an extreme value when chromium addition was 1810 μg/kg (Fig 5). Previous studies also indicated that broilers fed 2000 μg/kg chromium had greater ADG than those fed lower chromium concentrations (400 μg/kg) [17]. In fact, recent studies have demonstrated that the beneficial effects of chromium require high, pharmacologically relevant doses, far in excess of nutritionally relevant doses [12, 43]. However, further work is still required to evaluate the safety and effect of high doses of CrPic supplementation on broilers, which is of great significance to the practice of the broiler industry.

Meta-analysis is greatly influenced by the literature sources. Excluding languages other than English may introduce a language bias and excluding grey literature sources such as conference proceedings, government reports and pre-prints articles may introduce a publication bias, in which both may lead to erroneous conclusions [44]. In addition, there was a significant publication bias in ADG studies, although the sensitivity analysis indicated that the results of the meta-analysis showed low sensitivity and high stability (Figs 6–8). Sterne et al. suggested that Egger’s test based on fewer than 20 studies was generally considered to be inappropriate and had low sensitivity in the meta-analysis [45]; thus, the reasons for publication bias in this study might derive from the small number of included studies. Some interesting conclusions were obtained from the subgroup analysis; however, it is worth noting that some subgroups contained only one study, which likely will not lead to robust results. Hence, our study may have some limitations; however, we hope that our meta-analysis may contribute to inspiring future research in the field of broiler growth performance.

In conclusion, chromium supplementation significantly promoted growth performance by improving the average daily gain of broilers. Through subgroup analysis, we found that male broilers and Ross 308 might be more sensitive to chromium picolinate and showed better growth performance. Our meta-analysis also demonstrated that the benefits of Cr supplementation were mostly found under stressful conditions. An organic chromium supplementation at 1810 μg/kg was recommended for maximum average daily gain in this model. However, this study still has some limitations, and we expect more experimental studies and systematic reviews to provide supports and explanations for the mechanisms of broiler growth performance to feed additives.

## Supporting information

S1 Checklist PRISMA 2009 checklist. (DOC)

S1 File PRISMA 2009 flow diagram. (DOC)

S2 File (ZIP)
