# Supplementary material for: Effects of dietary chromium picolinate supplementation on broiler growth performance: A meta-analysis
Source: PLoS One. 2021 Apr 6;16(4):e0249527. doi: 10.1371/journal.pone.0249527 (PMC8023458; doi:10.1371/journal.pone.0249527)
Supplement: S1 File — (DOC) [file pone.0249527.s002.doc]

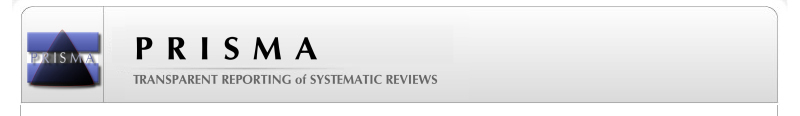
**PRISMA 2009 Flow Diagram**

**Screening**

**Included**

**Eligibility**

**Identification**

Records identified through database searching
(n =1330)

Additional records identified through other sources
(n =0)

Records after duplicates removed
(n = 1330)

Records screened
(n = 989)

Records excluded
(n =341)

Full-text articles assessed for eligibility
(n =115)

Full-text articles excluded, with reasons
(n =874)

Studies included in qualitative synthesis
(n =15)

Studies included in quantitative synthesis (meta-analysis)
(n =15)
